# Plerixafor in the Treatment of Stem Cell Mobilization Failure; First Experience in Iran 

**Published:** 2013

**Authors:** Mahshid Mehdizadeh, Abbas Hajifathali, Mahdi Tabarraee, Maria Tavakoli-Ardakani

**Affiliations:** a *Taleghani Bone Marrow Transplantation Center, Taleghani Hospital, Shahid Beheshti University Of Medical Sciences, Evin, Tehran, Iran. *; b *Hematology And Oncology Center, Shahid Beheshti University Of Medical Sciences, Tehran, Iran. *; c *Department of Clinical Pharmacy and Pharmaceutical Sciences Research Center, School of Pharmacy, Shahid Beheshti University of Medical Sciences, Tehran, Iran. *; d *Pharmaceutical Sciences Research Center, Shahid Beheshti University of Medical Sciences, Tehran, Iran. *

**Keywords:** Plerixafor, Mobilization, Stem cell transplant

## Abstract

High-dose chemotherapy and autologous stem cell transplantation (SCT) have become an effective care for many patients with hematological malignancies. Harvesting the stem cells is one the most important parts of SCT. The two most commonly used mobilization regimens are the use of granulocyte colony-stimulating factor (G-CSF) or G-CSF plus chemotherapy. However, about 10-30% of patients are unable to collect enough cells to support HSCT due to previous chemotherapies, radiation, marrow involvement or fibrosis. In multiple myeloma patients, it is hard to collect enough stem cells when the bone marrow is extensively involved. Plerixafor has emerged as a novel mobilizing agent and its efficacy has been shown in two phase III studies. Considering the importance of autologous SCT in patients with multiple myeloma, we report the first successful Iranian experience at Tehran Taleghani bone marrow transplantation center using plerixafor to mobilize stem cells in a patient with refractory multiple myeloma with extensive bone marrow involvement who failed mobilization with G-CSF.

## Introduction

High-dose chemotherapy and autologous stem cell transplantation (SCT) have become an effective care for many patients with hematological malignancies. For sure, harvesting the stem cells is one the most important parts of SCT (1-2). The two most commonly used mobilization regimens are the use of granulocyte colony-stimulating factor (G-CSF) or G-CSF plus chemotherapy (3). However, about 10-30% of patients are unable to collect enough cells to support HSCT after being mobilized with these regimens due to previous chemotherapies, radiation, marrow involvement or fibrosis (4). In multiple myeloma patients, it is hard to collect enough stem cells when the bone marrow is extensively involved. It seems that plerixafor may be effective. Plerixafor (AMD3100, Mozobil, Genzyme, USA) has emerged as a novel mobilizing agent and in two phase III studies, its efficacy in mobilizing patients with non- Hodgkin lymphoma (NHL) and multiple myeloma (MM) has been shown (4-6). Moreover, this agent demonstrated a safe profile with mild-to-moderate side effects, mainly gastrointestinal disorders and injection site reactions (5, 6). 

Considering the importance of autologous SCT in patients with multiple myeloma, we report our successful experience at Tehran Taleghani bone marrow transplantation center using plerixafor to mobilize stem cells in a patient with refractory multiple myeloma who failed mobilization with G-CSF.

*Case report*

A 50 years old female who had been diagnosed as multiple myeloma 8 months before the admission referred to BMT clinic for autologous SCT. In physical examination, she had fatigue, weakness, pallor and neuropathy (grade III in lower extremities and grade II in upper extremities). At the beginning of her illness, the results of laboratory tests were as follows: WBC = 3970/μL, Hb = 11.6 g/dL, platelet = 14000/μL (she had a history of chronic ITP since adolescence), ESR = 36 mm/h, LDH = 486, Ca = 10.1 mg/dL, urea = 26 mg/dL, Cr = 1.13 mg/dL, CRP = 6 mg/L. Her serum protein electrophoresis showed panhypogammaglobulinemia (IgG = 569 mg/dL, IgM = 11 mg/dL, IgA = 119 mg/dL) with a presence of a slightly dense monoclonal band in gamma region. Urine protein electrophoresis showed severe monoclonal proteinuria (11.7 g/24 h with 89.5% monoclonal protein) in gamma region. *β*_2_ micro globulin was 6.7 μg/mL. The patient had more than 75% plasma cells with many atypical forms in bone marrow biopsy. The skull x-ray showed lytic lesions. She received VAD regimen (Vincristine 0.25 mg/m^2^, Adriablastin 9 mg/m^2^ and Dexamethasone 40 mg/day for four days) plus Thalidomide for 4 courses with no response. Then, she received Bortezomib 1.3 mg/m^2^ (on day 0, 4, 8, 11) + Dexamethasone 40 mg/day for four days every 21 days. After 14 injections of Bortezomib, she had neuropathy grade III. The patient evaluation after second regimen chemotherapy showed an abnormal monoclonal band in gamma region in serum and 710 mg protein mostly (59.7%) monoclonal in gamma region in 24 h urine. *β*_2_ Micro globulin was 7.24 μg/mL. Her bone marrow showed more than 50% plasma cell again. She was considered as a refractory multiple myeloma and was prepared for autologous SCT. Just before the patient’s admission for SCT, her bone marrow biopsy showed 70-80% plasma cells. She received the first G-CSF 10 μg/Kg/day for five days but it was stopped due to no increment in WBC count. Then, the patient underwent chemotherapy with 2 g/m^2^ of Cyclophosphamide and 40 mg/day Dexamethasone for 4 days and G-CSF 10 μg/Kg/day (startrd on 6^th^ day). Two weeks later, when WBC count reached 5000/μL she received Plerixafor 0.24 mg/Kg 11 h and 0.15 mg/Kg (totally one vial) 6 h before the first apheresis. She underwent apheresis during 2 consecutive days with Cobe spectra apheresis devise and 4.07 × 10^8^/Kg mononuclear cells (MNC) were harvested (First day 2.36 × 108 MNC/Kg and second day 1.71 × 10^8^ MNC/Kg). She received Melphalan 200 mg/m^2^ and stem cells were infused as bone marrow rescue. She engrafted neutrophils on day +8 and platelet on day +15. During the hospitalization, the patient developed neutropenic fever that lasted 6 days, grade III mucositis and hemorrhagic cystitis due to BK virus activation on day +7, all treated based on BMT ward protocols. All blood cultures, serial *Galactomannan test *and CMV PP65Ag were negative.

The bone marrow aspiration on day +20 showed moderate hypocellularity with normal hematopoiesis and less than 5% plasma cells. She was discharged from hospital on day +24.

## Discussion

Clinical trials showed that high-dose chemotherapy followed by autologous SCT results in an increased complete response rate as well as higher overall survival in patients with multiple myeloma (4). In this case, due to the massive bone marrow involvement, we couldn’t harvest enough stem cells but after using combination of plerixafor, G-CSF and chemotherapy, we could collect enough cells and she underwent transplantation successfully. In clinical trials, plerixafor, in combination with G-CSF, increased the number of patients achieving both the minimum and target stem cell levels in fewer apheresis sessions (4, 6).

In a study in 2011, the addition of plerixafor to G-CSF in 56 patients with previous mobilization failure leaded to harvest enough stem cells in more than 75% of patients. In another study on 13 patients with multiple myeloma and lymphoma, it has been demonstrated that adding plerixafor to G-CSF after chemotherapy is a safe method for mobilization. In all 13 patients, enough cells have been collected, five from thirteen underwent transplant. In multiple myeloma cases, mean neutrophil and platelet engraftment time were on day +12 (7). We had also early engraftment in proper time on day +8 and +15 in our patient. The complications in this patient were also the same as other autologous transplants. There are not so many studies regarding mobilization in multiple myeloma patients with massive bone marrow involvement. In our case despite massive bone marrow involvement, we successfully harvest enough stem cells. It seems that in addition to the patients in whom it is hard to collect the stem cells due to several chemotherapies or myelotoxic agents, in patients with massive bone marrow involvement, plerixafor may also be effective. The influence of this drug has also been reported for mobilization in patients with other malignancies like germ cell tumor (8).

So it seems that even in multiple myeloma patients with massive bone marrow involvement, it is possible to collect enough stem cells by using Plerixafor and this medication is effective in patients refractory to common therapeutic methods for mobilization.
